# A biocompatible synthesis of gold nanoparticles by Tris(hydroxymethyl)aminomethane

**DOI:** 10.1186/1556-276X-9-220

**Published:** 2014-05-07

**Authors:** Feng Chen, Yanwei Wang, Jun Ma, Guangcan Yang

**Affiliations:** 1 School of Physics and Electronic Information Engineering, Wenzhou University, Wenzhou 325035, China

**Keywords:** Gold nanoparticle, Tris(hydroxymethyl)aminomethane, Biocompatible

## Abstract

Gold nanoparticles' novel properties are widely realized in catalysis, plasmonics, electronics, and biomedical applications. For biomedical application, one challenge is to find a non-toxic chemical and/or physical method of functionalizing gold nanoparticles with biomolecular compounds that can promote efficient binding, clearance, and biocompatibility and to assess their safety to other biological systems and their long-term effects on human health and reproduction. In the present study, we describe a new method by using Tris(hydroxymethyl)aminomethane (Tris), a widely used buffer solvent of nucleic acid and proteins, as the reducing agent for synthesizing gold nanoparticles by one step. It is found that Tris carries out the reduction reactions in relatively mild conditions for biomacromolecules. Particularly, it can be used to modify the DNA during the process of preparation of gold nanoparticles. The morphology and size distribution of gold nanoparticles are consistent and were confirmed by many different approaches including dynamic light scattering (DLS), UV-visible (UV-vis) spectrophotometry, atomic force microscopy (AFM), and transmission electron microscopy (TEM).

## Background

Chemical and physical properties of gold nanoparticles are dependent of their sizes, shapes, and crystallinity [1]. Up to now, most of the protocols for preparing gold nanoparticles (AuNPs) focus on the particles whose diameters range from 2 to 200 nm with various morphologies [2-5]. Due to their electrochemical properties, such as high affinity with biomolecules and their well-known optical absorption in the visible region surface plasmon band (SPB), gold nanoparticles are proved to be ideal nano-objects for medical imaging and even for photo-thermal therapy [6-8]. Therefore, the application of AuNPs in the biomedical field is growing exponentially. Despite a variety of reductants have been used to stabilize and synthesize AuNPs, only three approaches have been explored to produce size-defined gold nanoparticles through chemical reduction for medicinal applications. They are the citrate capping method [9], the biphasic Schiffrin-Brust method, and the seeding growth method [10,11]. Reducing agents or stabilizers and synthetic processes under non-toxic chemicals are important for biocompatible application, particularly for additional integration of the nanoparticles with other biological substrates, which is useful in diagnostic procedures, drug delivery, therapies, and biomedical applications [12]. Thus, a biocompatible protocol with a direct one-pot reaction in a mild condition and well-controlled shapes and sizes is needful in these fields. Many biomolecules such as liposomes [13], plant extracts [14], and chitosan [15,16], as stabilizer and/or reducing agents, have been directly used to synthesize AuNPs. In the present investigation, we describe a new method of designing and synthesizing gold nanoparticles by using Tris(hydroxymethyl)aminomethane. As we have known, Tris is one of the most widely used buffers of nucleic acids and proteins in biochemistry and biotechnology, and has also been adopted as a ligand for the synthesis of chromatographic adsorbents [17,18]. It is quite active in reduction reactions in various conditions due to its specific structure. More importantly, the reducing agent makes it possible to modify DNA during the process of preparing AuNPs. This feature is very useful for some applications, such as sensors, spectroscopic enhancers, quantum dot, nanostructure fabrication, microimaging methods, and ultrasensitive detection [19-22].

## Methods

Gold nanoparticles were synthesized by Tris method. Chloroauric acid (AR; 97 ml, 0.01% *m*/*m*, SCRC, Beijing, China) with 4 ml Tris solution was stirred using a magnetic stirrer, reduced to Au(0) slowly at 600 rpm at 40°C, and remained for 10 min until it has no visual change for another 10 min. Then, 3 ml NaOH (pH > 14) solution was injected to the solution drop by drop, meanwhile increasing the temperature of the solution to 50°C slowly. We could see the color of the solution gradually change from pink to deep red wine color. After about 8 min, the temperature was decreased to room temperature while stirring was continued to cool the solution. The reaction solution was then centrifuged at 12,000 rpm for 20 min (Xiang Yi centrifugal machine, Changsha, China), and its supernatant was removed; then, the AuNP solution was diluted to the original concentration with ultrapure water (18.2 MΩ, produced by a Milli-Q system, Millipore Co., Billerica, MA, USA). Absorptions were measured using a UV-2450 spectrophotometer (Shimadzu Co., Nakagyo-ku, Kyoto, Japan) operated at a resolution of 1 nm. Atomic force microscopy imaging was performed on SPM-9600 (Shimadzu Co.). Samples for transmission electron microscope (TEM) analysis were prepared by dropping Au nanoparticle solutions onto carbon-coated copper grids. JEM-2100 F (accelerating voltage 200 kV, JEOL, Ltd., Akishima, Tokyo, Japan) was used for obtaining the TEM images, and dynamic light scattering (Nano-zeta-size 90, Malvern Instruments, Westborough, MA, USA) was used mainly for the measurement of particle size and zeta potential.

## Results and discussion

### UV-vis spectroscopy and atomic force microscopy

**Figure 1:**
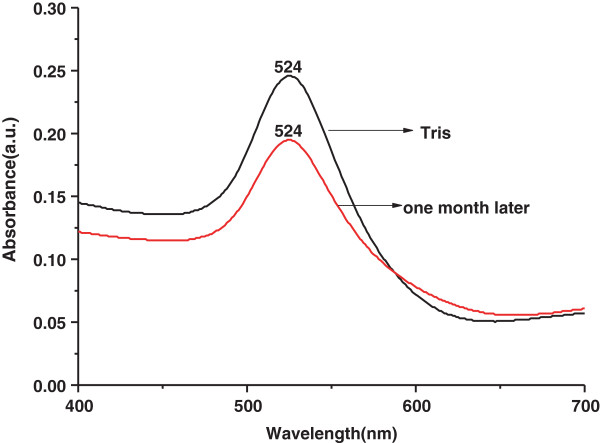
**UV-vis spectra for AuNPs synthesized by Tris.** The black line corresponds to the fresh AuNP UV-vis extinction curve, and the red line is the spectra of the same sample stored for a month.

**Figure 2:**
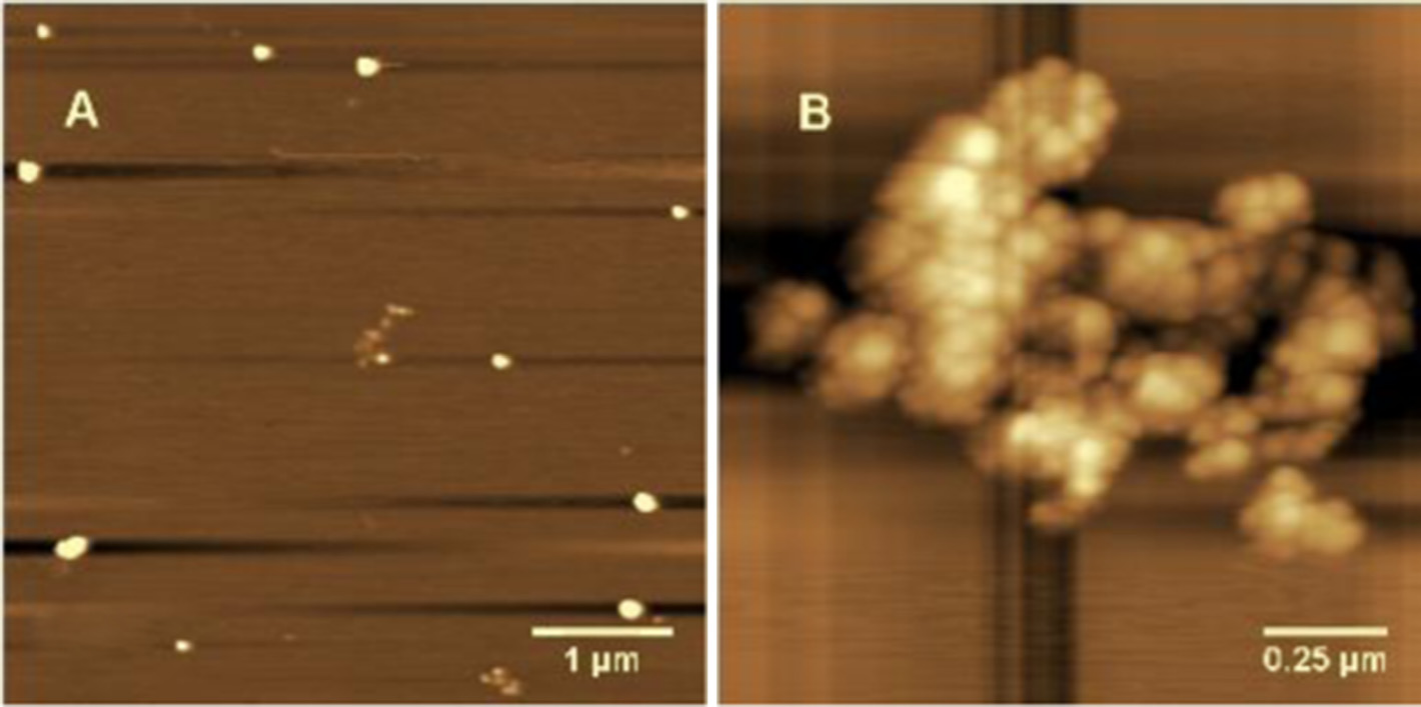
**AFM image of AuNPs synthesized by Tris. (A)** Fresh sample dropwise added on the mica, and **(B)** the same sample stored for 2 days, in which the nanoparticles were assembled.

The UV-visible (UV-vis) light absorption pattern was kinetically monitored in the range of 300 to 800 nm. Figure 1 shows the UV-vis absorption spectra of the gold nanoparticle solution after 2 h of reaction (black line). The results indicate that the reaction solution has an absorption maximum at approximately 524 nm, a feature shift typical for spherical AuNPs. According to the Mie theory, the purification process removed the low-molar-mass impurities without fundamentally altering the structure of the nanoparticles (NPs) [23]. It has been already known that colloid particles tend to agglomerate slowly *in vitro* in the previous study. Atomic force microscopy (AFM) images showed that Tris-prepared gold nanoparticles agglomerated to form a layer more easily, when the specimens were placed on a mica plate over 48 h (Figure 2). Nevertheless, if stored at 4°C, the gold nanoparticles can maintain stable with its intrinsic properties more than several months; in other words, the particles still remain their dispersities and structural properties. The ultraviolet-visible spectrum of the Au solution is still peaked at 524 nm only with a little decrease of the absorption intensity (Figure 1, red line).

### The images of transmission electron microscope

**Figure 3:**
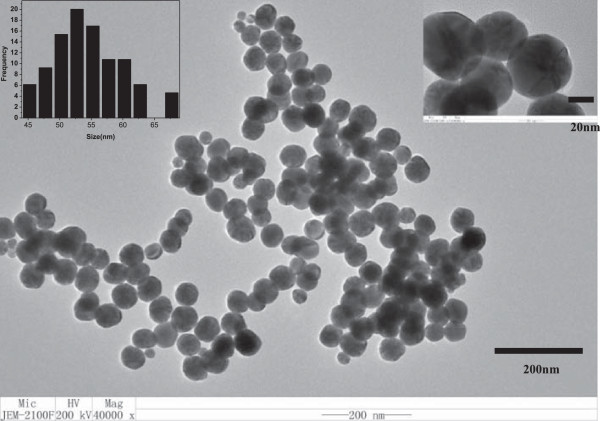
**TEM images of gold nanoparticles.** Gold nanoparticles synthesized by Tris. The insets show higher magnification images and size distribution histogram. The gold nanoparticles have perfect polyhedral structures and nanoparticle diameter = 55.2 ± 8.0 nm.

Figure 3 shows the TEM images of the gold nanoparticle colloid solution reduced by Tris. We can notice that all of the AuNPs have similar icosahedral structures and are monodisperse with a mean diameter approximately equal to 59 nm. Although there are some overlaps among parts of gold nanoparticles, they are not agglomerated; instead, the morphology of each particle is still maintained. Since the UV characteristic peak of the particle solution can maintain for a very long time, we infer that the particles are just more easy to self-assemble into a membrane to avoid agglomeration.

**Figure 4:**
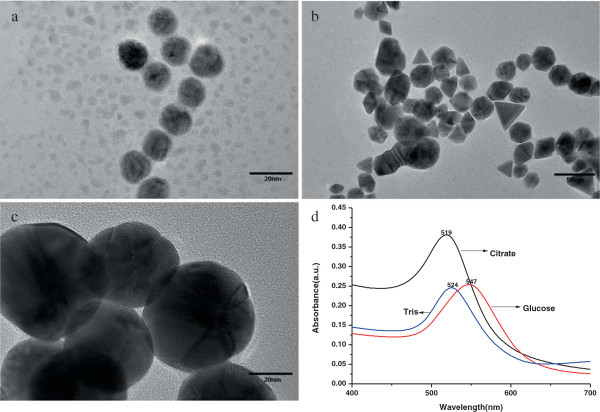
**TEM and UV-vis images. (A)** Gold nanoparticles by Tris. **(B)** Nanoparticles by glucose form a variety of structures. **(C)** Gold nanoparticles by sodium citrate are spherical monodispersed. **(D)** The UV-vis spectra: the absorbance intensity of AuNPs by citrate methods, glucose method, and Tris method are peaked at 519, 547, and 524 nm, respectively.

The development of Tris method is inspired by the classic citrate method and the ‘greener’ glucose method for gold nanoparticle preparation. Hence, we used citrate- and glucose-reducing gold nanoparticles for comparison with Tris in the current experiments. The 4 ml solution of 1% sodium citrate, Tris, and glucose with the same mass ratio of the concentration were added, respectively, to three parts of 97 ml chloroauric acid solution of concentration 0.01% (in which the volume with trisodium citrate gold solution was 100 ml, since the reaction does not require adding the alkaline solution). The color of the final gold nanoparticle solution by trisodium citrate solution is blood red, the one by glucose is purple, while gold colloid by Tris solution looks like a red wine. After the centrifugal process mentioned above, we measured the ultraviolet absorption spectrum of the three kinds of colloidal solutions. Their spectroscopies showed that the absorption peaks distributed at 519 nm (citrate), 524 nm (Tris), and 547 nm (glucose) (Figure 4D), respectively. We can see that the absorption peaks of colloidal solutions by glucose and Tris methods appear redshift to the one by citrate. As we have known, the gold nanoparticles prepared by trisodium citrate are typically spherical, while nanoparticles by Tris and glucose may have different shapes and sizes deduced from their redshift peaks. In order to clarify these features further, we used the dynamic light scattering (DLS) to measure the size distributions of the three kinds of gold nanoparticles extensively. The results are shown in Table 1, where we can see that the average diameters of these particles are 12.9, 23.79, and 59.29 nm, respectively. These features are confirmed further by TEM, as shown in Figure 4. In Figure 4A, we can see that the nanoparticles by sodium citrate basically are spherical and monodisperse with diameters around 13 nm. However, the particles prepared by glucose have various shapes, as shown in Figure 4B. It seems that their shapes are dependent on the detailed reaction process. If we put much more 1 ml of NaOH solution and inject it faster into the solution, the high pH environment by the rapid supply of NaOH leads to the instantaneous formation of small nanoparticles and ends the reduction rapidly [12]. Therefore, the size and morphology of these gold nanoparticles depend strongly on the adding rate of NaOH. Among the three methods, we can see that the AuNPs by Tris are monodisperse with diameters around 60 nm, which are significantly larger than the other two, and appear as a perfect polyhedral structure as shown in Figure 4C. Actually, large polyhedral nanoparticles can find more applications in biomedical fields. For example, AuNPs can strongly adsorb thiol-capped DNA, depending on their large specific surface area and high surface free energy, and AuNPs with a diameter larger than 30 nm are more stabilized and essential for many diagnostic applications [20,24]. Our Tris-prepared nanoparticles are ideal candidates for these applications.

**Table 1:** Dynamic light scattering (DLS) data of the size distributions of three kinds of gold nanoparticles

**DLS**		**5 min**	**10 min**	**15 min**	**20 min**	**25 min**	**Average *d *** **(nm)**
Citrate	*d* (nm)	13.15	12.60	13.05	12.58	13.10	12.90
	*I* (%)	100	100	98.8	97.8	98.6	
Glucose	*d* (nm)	23.95	23.91	23.64	23.53	23.94	23.79
	*I* (%)	87.2	86.0	86.6	87.6	88.3	
Tris	*d* (nm)	57.63	62.42	57.37	57.63	61.40	59.29
	*I* (%)	92.2	96.2	95.9	95.7	93.4	

*I* represents the percentage of the main AuNPs in total particles.

For DNA conjugate application, we can further optimize the experimental process to make the reaction conditions much more mildly and to keep the DNA intact under such environment. For this purpose, we replaced the magnetic stirring by ultrasonic vibration, and the heating temperature was set to 45°C. First, the chloroauric acid solution was treated by ultrasonic and heated for 15 min (actual temperature is about 30°C). Then, the mixed solution of 2 ml of 10 mM Tris containing DNA at a concentration of 1 ng/μl with 2 ml NaOH whose mass fraction is 1% was added, and then the solution was ultrasonically treated for one more hour. The final DNA gold nanoparticles were ready for further conjugation.

**Figure 5:**
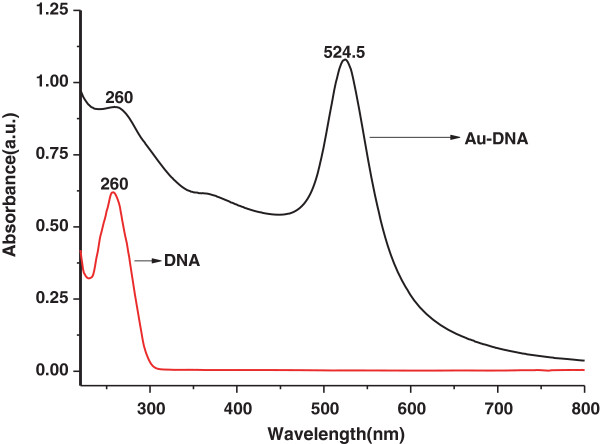
The UV-vis spectrum of Au-DNA and DNA solution.

We compared the measured zeta potential and UV-vis spectra (monitored in the range of 200 to 800 nm) of the Au-DNA to the DNA solution and found that the features of DNA (the zeta potential is −36.0 and a shoulder UV peak at 260 nm [25]) remain unchanged before and after the reaction (shown in Table 2 and Figure 5). Thus, the present preparation provides a new biomolecule-compatible example for biomedical applications.

**Table 2:** Zeta potential and mobility of pure DNA and gold nanoparticles prepared using Tris-DNA mixture at 25°C

	**Zeta potential (mV)**	**Mobility**
Pure DNA	−36.5	−2.858
Au-DNA	−36.0	−2.824

## Conclusion

In summary, we presented a new biocompatible synthesis method of gold nanoparticles by Tris, a widely used buffer of nucleic acids and proteins. The method has a useful feature to allow modifying the DNA during the process of preparation of gold nanoparticles. However, the mechanism responsible for biomolecule-directed gold nanoparticle formation remains unclear due to the lack of structural information about biological systems and the fast kinetics of biomimetic chemical systems in solution [26].

Tris(hydroxymethyl)aminomethane, or 2-amino-2-(hydroxymethyl)-1,3-propanediol, coordinates with a number of metallic ions. In order to understand its unusual acid-base and redox activities, we added Tris solution to chloroauric acid solution until it is completely mixed with the gold ions. Then, we slowly heated the solution to 40°C while adding NaOH solution gently into the solution until the color is stable, immediately adjusting the temperature to room temperature by stirring the solution. In the whole process, temperature does not exceed 45°C, and the stirring speed is about 1,000 rpm or less. In this protocol, by using Tris in alkaline aqueous solution, we can prepare large Au nanoparticles with a polyhedral structure in a temperately process. DNA or cells *in vitro* are capable of maintaining their intrinsic characteristics under such mild conditions. By using ultrasonic vibration, we can even prepare one-pot DNA-gold nanoparticle conjugates directly, ready for further applications of sensing, imaging, and ultrasensitive detection in biomolecular field [27-29]. During the reaction, a high concentration of NaOH solution is used to promote the reaction rate.

## Competing interests

The authors declare that they have no competing interests.

## Authors' contributions

FC and GY designed the study, analyzed the data, and wrote the manuscript. FC performed experiments. YW and JM contributed to the discussions and analytical tools. All authors read and approved the final manuscript.
